# Synthesis and Evaluation of Functionalized Polyurethanes for pH-Responsive Delivery of Compounds in Chronic Wounds

**DOI:** 10.3390/gels9080611

**Published:** 2023-07-28

**Authors:** Zhongyan Li, Matthew Crago, Timothy Schofield, Haoxiang Zeng, Heema Kumari Nilesh Vyas, Markus Müllner, Anne Mai-Prochnow, Syamak Farajikhah, Sina Naficy, Fariba Dehghani, Sepehr Talebian

**Affiliations:** 1 Faculty of Engineering, School of Chemical and Biomolecular Engineering, The University of Sydney, Sydney, NSW 2006, Australia; zhongyan.li@sydney.edu.au (Z.L.); matthew.crago@sydney.edu.au (M.C.); timothy.schofield@sydney.edu.au (T.S.); heema.vyas@sydney.edu.au (H.K.N.V.); anne.mai-prochnow@sydney.edu.au (A.M.-P.); syamak.farajikhah@sydney.edu.au (S.F.); fariba.dehghani@sydney.edu.au (F.D.); 2 Key Centre for Polymers and Colloids, School of Chemistry, The University of Sydney, Sydney, NSW 2006, Australia; hzen8512@sydney.edu.au (H.Z.); markus.muellner@sydney.edu.au (M.M.); 3 Sydney Institute for Infectious Diseases, The University of Sydney, Sydney, NSW 2006, Australia; 4 Nano Institute (Sydney Nano), The University of Sydney, Sydney, NSW 2006, Australia

**Keywords:** chronic wound dressing, controlled drug release, pH responsive hydrogels, polyurethane-based hydrogels

## Abstract

Chronic wounds, depending on the bacteria that caused the infection, can be associated with an extreme acidic or basic pH. Therefore, the application of pH-responsive hydrogels has been instigated for the delivery of therapeutics to chronic wounds. Herein, with the aim of developing a flexible pH-responsive hydrogel, we functionalized hydrophilic polyurethanes with either cationic (polyethylene imine) or anionic (succinic anhydride) moieties. A comprehensive physicochemical characterization of corresponding polymers was carried out. Particularly, when tested in aqueous buffers, the surface charge of hydrogel films was closely correlated with the pH of the buffers. The loading of the cationic and anionic hydrogel films with various compound models (bromophenol blue; negatively charged or Pyronin Y; positively charged) showed that the electrostatic forces between the polymeric backbone and the compound model will determine the ultimate release rate at any given pH. The potential application of these films for chronic wound drug delivery was assessed by loading them with an antibiotic (ciprofloxacin). In vitro bacterial culturing was performed using *Staphylococcus aureus* (*S. aureus*) and *Escherichia coli* (*E. coli*). Results showed that at the same drug dosage, different release profiles achievable from cationic and anionic polyurethanes can yield different degrees of an antibacterial effect. Overall, our results suggest the potential application of cationic and anionic hydrophilic polyurethanes as flexible pH-responsive materials for the delivery of therapeutics to chronic wounds.

## 1. Introduction

A significant global health challenge is posed by chronic wounds, such as diabetic foot ulcers (DFUs), venous leg ulcers, and pressure ulcers (PUs) [1]. The incidence of non-healing chronic wounds has exhibited a remarkable increase due to factors such as population aging and the concomitant growth in comorbidities like diabetes, venous insufficiency, extended hospitalization, and associated chronic diseases. Chronic wounds are thought to affect 2% among the general population, contributing to an annual financial burden exceeding USD 50 billion, with projections indicating a further escalation [2]. These chronic wounds, characterized by a prolonged duration ranging from months to years, render the wound bed susceptible to colonization by opportunistic infections, thereby exacerbating the patients’ conditions [3]. The colonization of wounds by bacteria is often facilitated by the formation of a slimy extracellular matrix, commonly known as biofilm, which is believed to impact approximately 80% of chronic wounds. Notably, approximately half of all DFUs are infected, and infection status is significantly linked to the half of all DFUs that are classified as infected, and this infection status strongly correlates with the need for amputation and an increased risk of mortality [4]. Pharmaceutical intervention represents a highly effective approach in the clinical management of chronic wounds, exerting direct influence on the wound healing process [5,6]. For wound treatment, several drug delivery strategies, such as oral administration, intramuscular injection, and localized exterior application, have been used in clinical use [7]. Notably, temporary wound dressings that serve as drug reservoirs have emerged as a promising strategy, enabling a sustained local drug release [8,9]. This localized drug delivery approach allows direct contact between the drugs and the wound site, minimizing their systemic circulation [10]. Consequently, the potential toxic side effects associated with systemic drug administration can be significantly mitigated. Leveraging these inherent advantages, substantial efforts have been dedicated to advancing the field of biomaterial-based drug delivery dressings, specifically tailored for both acute and chronic wound management [11].

Among various biomaterials, hydrogel dressings have garnered significant attention in the field of wound healing due to their remarkable properties: (1) Hydrogels are polymer materials that have the extraordinary capacity to absorb substantial quantities of water, creating a three-dimensional network structure. This feature produces the optimal moist environment within the wound site, which is essential for decreasing tissue necrosis and speeding up the growth of new epithelial tissue. (2) Hydrogels’ structural similarity to the extracellular matrix (ECM) offers a key benefit by allowing them to regulate the release of functional payloads. This characteristic allows for precise control over the delivery of bioactive agents and facilitates the maintenance of cellular activity within the wound microenvironment. (3) Hydrogels exhibit the ability to effectively absorb wound exudates, which aids in wound healing by promoting a favourable environment for gaseous exchange [12,13,14,15,16].

Hydrogel dressings enabled the successful distribution of medicines into the wound bed at the appropriate dosage and time. Particularly, pH-responsive hydrogels have emerged as ideal candidates for drug delivery in chronic wounds, given the wound pH level is a significant signal for monitoring the healing progress [17,18]. However, pH-sensitive hydrogel dressings have a number of drawbacks despite being affordable and practical, including poor mechanical strength, challenges with medication loading, and local hazardous responses. Additionally, due to their uneven healing rates, chronic wounds frequently show a spatial irregularity of infection development, resulting in major pH variations through the region of infection and necessitating specific management over the release pattern of medications to avoid high-dose-generated adverse side effects [19].

Hydrophilic polyurethanes (HPUs), made up of di-cyclohexyl isocyanate (DCHI) hard segments and polyethylene glycol (PEG) soft segments of a varied chain length, are a type of hydrogels that are mainly crosslinked by hydrogen bonds, providing mechanical recoverability, toughness, and customizable physical and mechanical qualities [20]. Our team previously explored the application of HPU in a variety of biomedical applications including but not limited to artificial heart valves [21], cell transplantation scaffolds [22,23], biosensors [24,25], and bioactuators [20,26,27,28,29]. For the development of pH-responsive drug delivery systems, acid-cleavable bonds such as hydrazone bonds, acetal linkage, and ortho-esters have been implemented into the structure of polyurethane [30]. However, one of the main disadvantages of these labile bonds is their irreversible nature after dissociation. This is critical for drug delivery to chronic wounds, as the pH value can fluctuate during the healing process. To this end, the functionalization of polyurethanes with either amine or carboxylic acid groups was suggested as an alternative to establish pH-sensitive drug delivery systems. For instance, amphiphilic poly(ether urethane) containing triblock copolymer Poloxamer^®^ 407, an aliphatic diisocyanate (i.e., 1,6 hexamethylene diisocyanate), and a cyclic chain extender (i.e., 1,4-cycloexhanedimethanol) was implemented [31]. Subsequently, the surface activation of the polymer was achieved using plasma treatment followed by the anchoring of the acrylic acid moiety on the polyurethanes. The carboxylic-acid-modified polymer was tested in vitro, and the obtained results showed a significantly higher amount of Ibuprofen (an anti-inflammatory drug) released in an alkaline pH (8) when compared to an acidic pH (5). This observation was assumed to be a consequence of the extreme swelling of hydrogels in the alkaline pH due to the deprotonation of carboxylic acid groups. A main disadvantage of this technique is the lack of control over the degree of functionalization, as well as polymer degradation due to plasma exposure.

**Figure 1:**
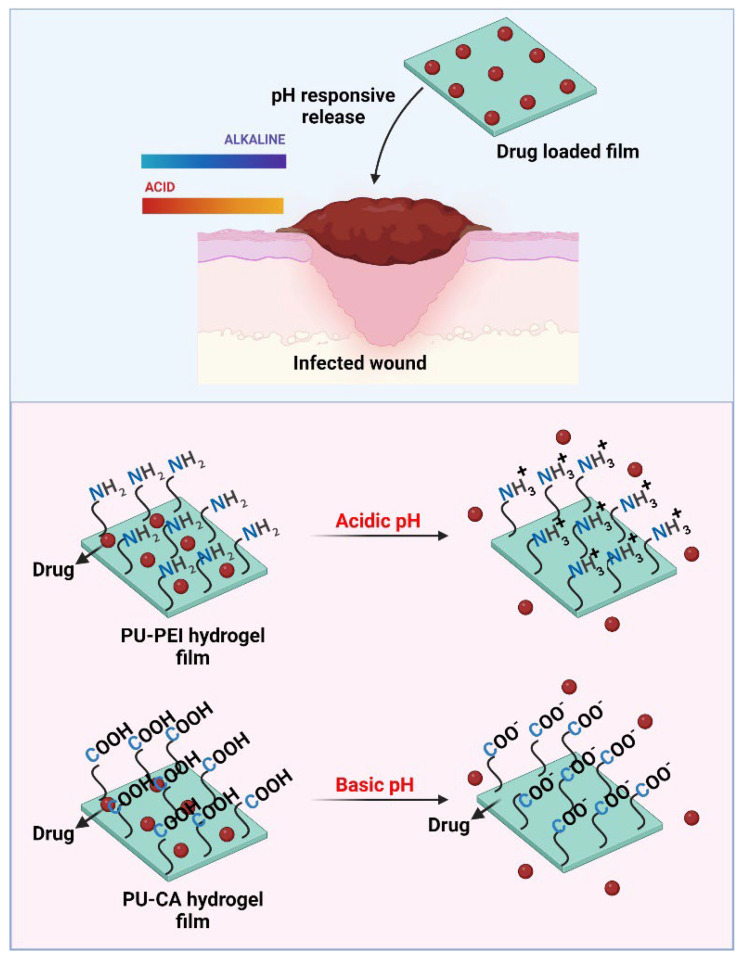
Graphic showing acidic or alkaline pH of an infected wound, and the design of pH-sensitive films for responsive release of drugs in these wounds.

In this study, for the first time, two different chemistries were introduced for the functionalization of HPU either with amine or carboxylic acid groups (Figure 1). Specifically, (i) an aminolysis mechanism to attach polyethylenimine (PEI) to the polymer backbone, and (ii) a Steglich esterification to decorate the polymer backbone with succinate acid. Subsequently, a comprehensive physicochemical characterization of the synthesized polymers was carried out to confirm the attachment of targeted moieties. Particularly, surface zeta potential measurements of the modified PU films showed that amine-modified films (PU-PEI) possess a highly positive charge at an acidic pH (3.5), which gradually diminishes with an increase in the pH value. On the other hand, carboxylic-acid-modified films (PU-CA) possessed no charge at an acidic pH, while they gradually gained more negative charge at a highly alkaline pH (9). The two types of HPU films were tested in vitro for the release of both positively and negatively charged model compounds (Pyronin Y and bromophenol blue, respectively). The results showed that regardless of compound model charge, PU-PEI films released more cargo in an acidic pH, while PU-CA films were releasing more in an alkaline pH. It is important to note that a step pH release test revealed the responsiveness of these films even after three cycles of pH changes. To test the suitability of these films for chronic wound drug delivery, they were loaded with ciprofloxacin (antibiotic for both Gram-negative and Gram-positive aerobic bacteria) and subsequently tested in vitro on *Staphylococcus aureus* (*S. aureus*) and *Escherichia coli* (*E. coli*). At the same drug dosages, PU-PEI films were capable of showing a significantly higher antibacterial effect as compared to the PU-CA films. These observations suggested that at a neutral pH of the culture media (7.4), PU-PEI films yielded a slower release profile, which instigated their enhanced antibacterial effect. Overall, the chemically modified HPUs showed potential to be used as wound dressings capable of releasing antibiotics in response to the pH of the infected wounds. 

## 2. Results and Discussion

### 2.1. Physicochemical Characterization of Synthesized Polymers

**Figure 2:**
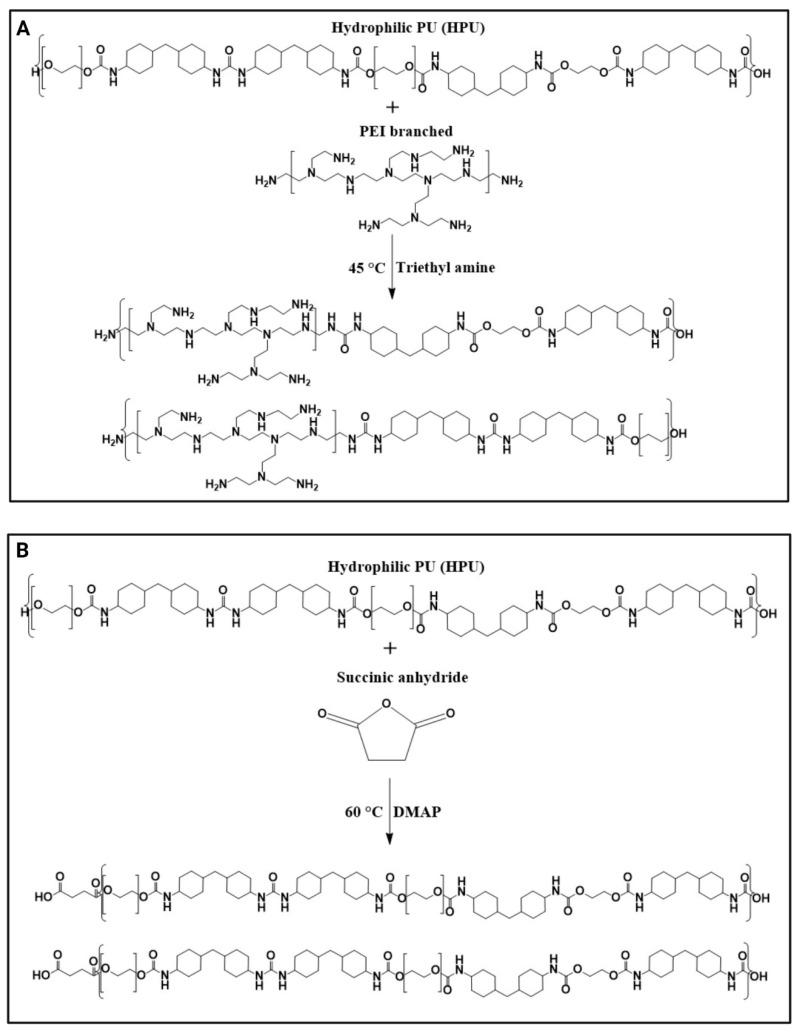
Schematics showing (**A**) synthesis of PU−PEI, (**B**) synthesis of PU−CA.

**Figure 3:**
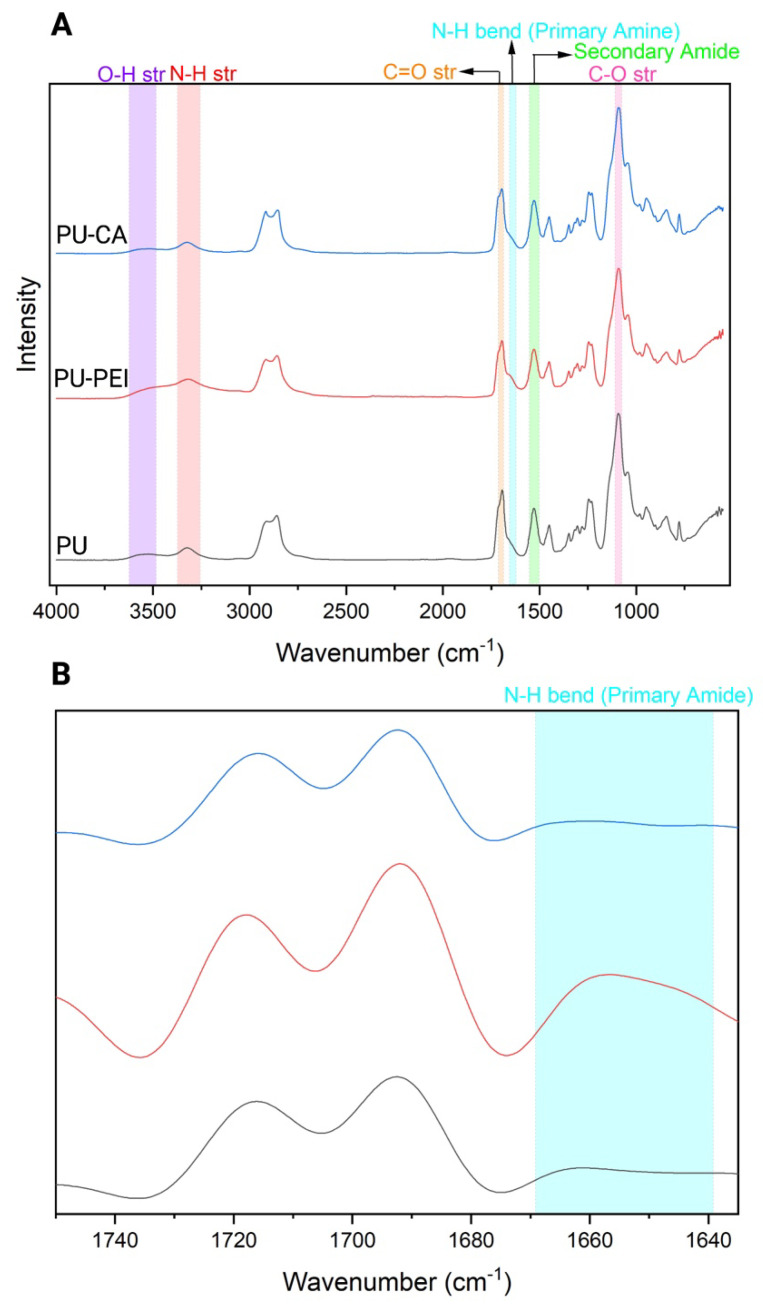
(**A**) FTIR spectra of PU, PU−PEI, and PU−CA; (**B**) deconvolution of FTIR spectra of PU, PU−PEI, and PU−CA in the region between 1640 and 1740 cm^−1^.

PU−PEI and PU−CA were synthesized using aminolysis and Steglich esterification methods, respectively (Figure 2). The FTIR spectroscopy was conducted to confirm the chemical structure of the following polymers (Figure 3A). Accordingly, PU characteristic bonds appeared at 1090, 1530, 3323, and 3547 cm^−1^ attributed to -C-O stretching, the -NH stretching of secondary amine, the -NH bending of secondary amine, and -OH stretching, respectively [29]. 

PU−PEI polymers showed a similar spectrum but the appearance of a shoulder at 1644 cm^−1^ (as confirmed with the deconvoluted spectrum; Figure 3B) was associated with the -NH bending of primary amines of the PEI backbone. Compared to PU, the PU−CA spectrum showed a more intense band at 1090 cm^−1^ associated with -C-O stretching, indicating the formation of ester bonds, and a less intense band at 3547 cm^−1^ associated with -OH stretching due to the consumption of these groups in the Steglich esterification. ^1^H NMR was utilized to further investigate the chemical structure of synthesized polymers (Figure S1). PU−PEI showed the addition of shifts between 2.5 and 3.25 ppm attributed to protons of PEI [32]. PU-CA showed a chemical shift between 2.65 and 2.75 ppm associated to methylene protons of succinic acid [33]. Furthermore, by using the peak heights of each element from XPS spectra (Figure S2), it was shown that PU−CA had a much higher O1s/C-C value (0.98) as compared to PU (0.28). On the other hand, PU−PEI had a much higher N/C-C value (0.14) when compared against PU (0.015). The obtained values further indicated the addition of amines and carboxylic acids to the corresponding PU−PEI and PU−CA polymers. 

**Figure 4:**
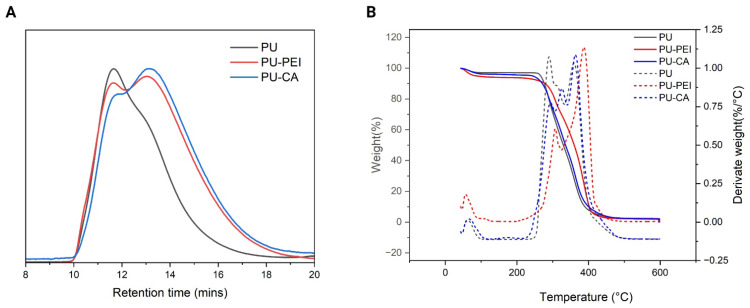
(**A**) SEC of PU, PU−PEI, and PU−CA; (**B**) TGA and DTGA of PU, PU−PEI, and PU−CA.

The SEC analysis was performed to measure the weight average molecular weight (M_W_) of the synthesized polymers (Figure 4A). The results revealed that PU possessed an M_W_ of 332,300 Da while this value for PU−PEI was 267,200 Da. The decrease in the M_W_ was due to the scrambling of the PU backbone due to the amylosis reaction. Likewise, PU−CA polymers showed a decrease in M_w_ to 223,100 Da. This observation was assumed to be likely due to hydrolysis or transesterification in the backbone of PU. Furthermore, the TGA analysis was conducted and the results for PU exhibited three steps of decomposition. The first step was attributed to the decomposition of urethane bonds; the second and third were considered as consecutive and related to the decomposition of ester groups (Figure 4B) [34]. PU−CA showed a similar trend; however, PU−PEI only had two decomposition bands with the urethane decomposition peak being a lot less intense, probably due to the aminolysis reaction mechanism. Lastly, the rheology of the polymers was studied using the shear test and oscillatory experiments, and no significant difference was observed between the viscosity, E′, and E″ of the three polymers (Figure S3). It was hypothesized that hydrogen bonds in PU−PEI and PU−CA nullified the effect of the slight decrease in the molecular weight of the polymers. 

### 2.2. Physicochemical Characterization of Hydrogel Films

**Figure 5:**
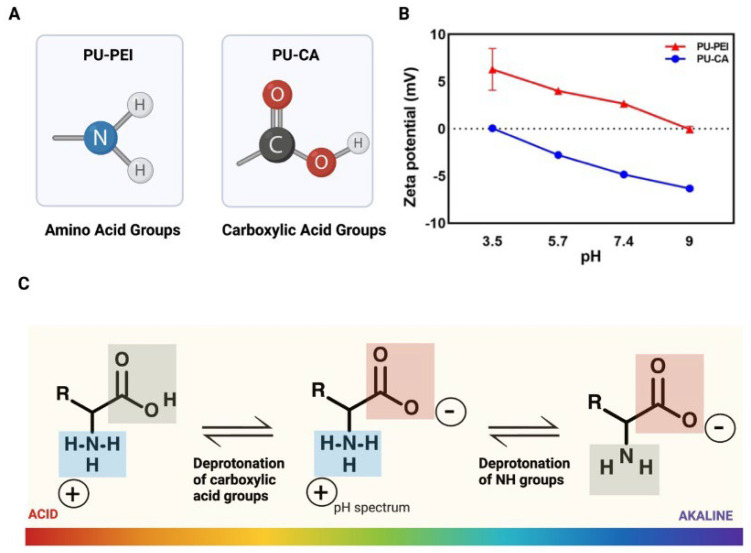
(**A**) Schematic of the majority of groups found in PU−PEI and PU−CA hydrogels; (**B**) surface zeta potential measurements of PU−PEI and PU−CA hydrogel films; (**C**) schematic of the primary charges of amino acid and carboxylic acid groups across the pH scale.

The surface charge of PU−PEI and PU−CA hydrogel films was measured with SurPass3 (Anton Parr) at pH (3.5), (5.7), (7.4), and (9) (Figure 5B). At pH (3.5), PU−PEI hydrogel films shift towards positive zeta potential values (7.4 ± 1.1), which is due to the protonation of amino groups from polymers [35]. Carboxylic acid groups start to become deprotonated as pH increases towards more basic values (Figure 5C). As a result, the zeta potential of PU−CA dropped from nearly neutral to −6.3 ± 0.1 as pH was raised from (3.5) to (9). These observations further revealed that the protonation and deprotonation of corresponding functional groups will dictate the surface charge of these films. 

**Figure 6:**
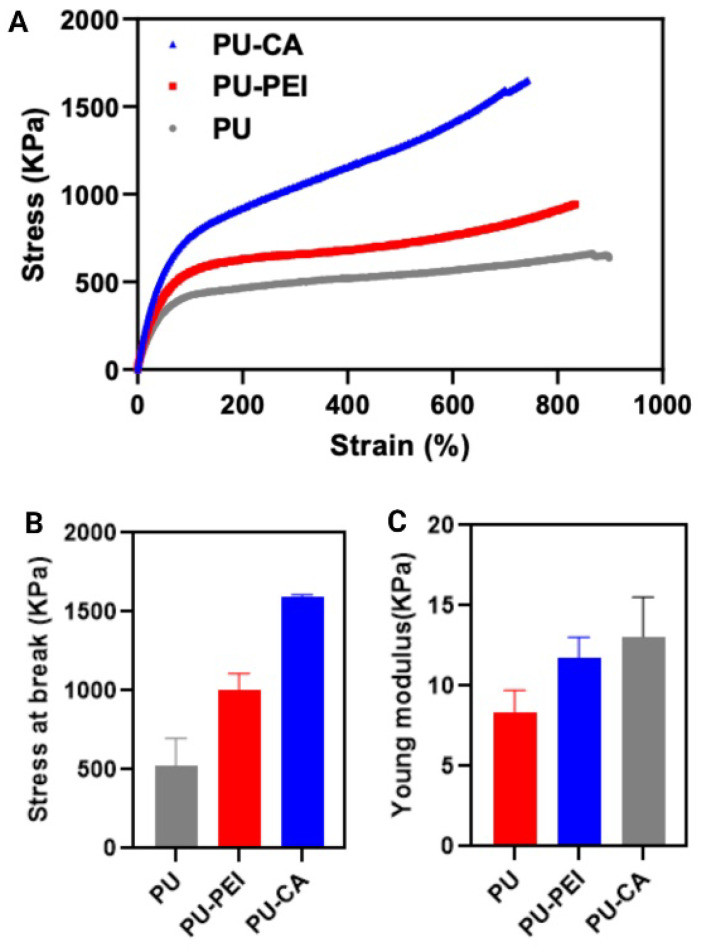
(**A**) Stress–strain behaviour of PU, PU−PEI, and PU−CA hydrogel films; (**B**) stress at break of PU, PU−PEI, and PU−CA hydrogel films; (**C**) Young’s moduli of PU, PU−PEI, and PU−CA hydrogel films.

Next, mechanical properties of the films were measured (Figure 6). PU−CA exhibits an increase in both stress and Young’s modulus compared to PU and PU−PEI, which could be an indication of the possible crosslinking of the polymeric chains via succinic acid moieties. On the other hand, PU−PEI showed a slight increase in the strength and modulus compared to PU, probably due to the formation of new hydrogen bonds between amine groups. The reduction in the molecular weight of the polymer (as measured with SEC) could also have some reducing effect on the mechanical properties when compared to PU−CA. The swelling ratio of all films was also tested at pH (3.5), (5.7), and (7.4) for PU−PEI hydrogel films and pH (5.7), (7.4), and (9) for PU−CA hydrogel films (Figure S4), and no significant difference was observed between swelling at these measured pHs. However, PU−PEI films possessed a higher swelling degree when compared to PU−CA, possibly due to a lower molecular weight of polymeric chains. 

### 2.3. In Vitro Release Study

#### 2.3.1. The Release of Model Compounds

**Figure 7:**
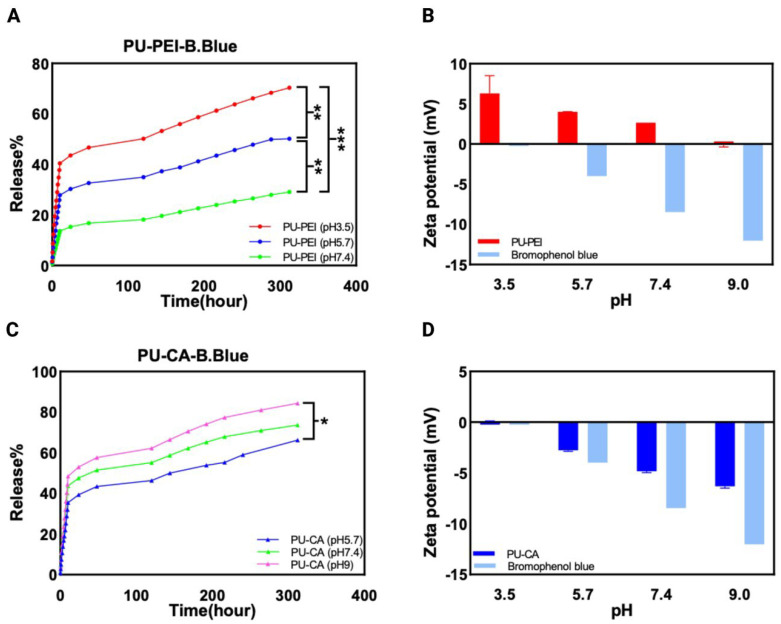
(**A**) The release profile of PU−PEI hydrogel film with bromophenol blue; (**B**) surface zeta potential measurements of PU−PEI hydrogel films and bromophenol blue; (**C**) the release profile of PU−CA hydrogel film with bromophenol blue; (**D**) surface zeta potential measurements of PU−CA hydrogel films and bromophenol blue. The *p*-value was used to determine the level of significance for data comparisons, with significance levels represented by asterisks (* *p* < 0.05, ** *p* < 0.01, *** *p* < 0.001).

Bromophenol blue and Pyronin Y were selected as the model compounds with a negative charge and positive charge (respectively) to assess controlled release in a changing pH environment. The first part of the in vitro study focused on the release profiles of bromophenol blue from PU−PEI hydrogels at pH (3.5), (5.7), and (7.4), with cumulative release percentages measured at various time intervals. The results revealed a substantial disparity in bromophenol blue release between different pH values, with higher release observed at lower pH levels. After 10 h, approximately 40% of bromophenol blue was released at pH (3.5) compared to around 13% at pH (7.4) (Figure 7A). Furthermore, as time advanced, the difference in release between pH values significantly increased, with the release at pH (3.5) being approximately three times higher than that at pH (7.4) after 120 h. These findings provide evidence for the increased release of bromophenol blue, as a negatively charged drug, from PU−PEI hydrogels in acidic environments, suggesting a pH-dependent release behaviour. This observation can be attributed to the weakened attractive interactions between the negatively charged bromophenol blue dye and the positively charged PU−PEI hydrogel film at highly acidic pH conditions (Figure 7B). The reduced attraction between the oppositely charged entities facilitates the diffusion and liberation of the dye molecules from the hydrogel matrix, leading to an enhanced release. This is further proved from swelling ratio measurements (Figure S2), as no significant difference was observed between swelling values of PU−PEI films at the measured pH of (3.5), (5.7), and (7.4). 

In contrast, we tested the release behaviour of bromophenol blue from PU−CA hydrogels at pH (5.7), (7.4), and (9). The cumulative release percentages after 10 h were approximately 37% at pH (5.7) and nearly 49% at pH (9) (Figure 7C). Notably, after 120 h, the release at pH (9) was approximately 1.5 times higher compared to the release at the lower pH values. This observation suggests that the release of bromophenol blue from PU−CA hydrogel films is augmented under basic conditions. This can be caused by the repulsive interactions between the negatively charged bromophenol blue dye and the negatively charged PU−CA polymer in alkaline environments (Figure 7D). The intensified repulsive forces further facilitate faster and higher diffusion of bromophenol blue molecules out of the polymeric network. This mechanism elucidates the pH-dependent release behaviour of bromophenol blue from PU−CA hydrogel films and highlights the crucial role of electrostatic repulsion in governing drug release kinetics in basic conditions. 

**Figure 8:**
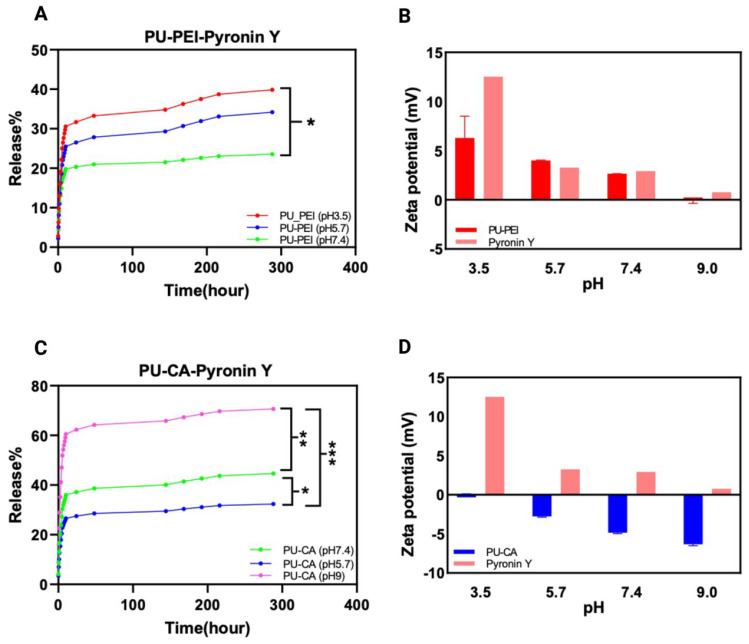
(**A**) The release profile of PU–PEI hydrogel film with Pyronin Y; (**B**) surface zeta potential measurements of PU–PEI hydrogel films and Pyronin Y; (**C**) the profile of PU−CA hydrogel film with Pyronin Y; (**D**) surface zeta potential measurements of PU−CA hydrogel films and Pyronin Y. The *p*-value was used to determine the level of significance for data comparisons, with significance levels represented by asterisks (* *p* < 0.05, ** *p* < 0.01, *** *p* < 0.001).

Next, we focused on using a positively charged compound model (Pyronin Y) to study the possible effect of its charge on the obtained release profile from the films. The results indicate that the release of Pyronin Y increases as the pH decreases at all measured time points (Figure 8A). Similar to negatively charged bromophenol blue with PU−CA hydrogels, this can be attributed to the strong repulsion between the positively charged Pyronin Y and the positively charged PU−PEI at a lower pH (Figure 8B). This repulsion disrupts the ionic interactions between Pyronin Y and the hydrogel, resulting in a higher release of Pyronin Y. Conversely, at higher pH levels, the repulsion is reduced, leading to a lower release of Pyronin Y. In the case of PU−CA hydrogel, the obtained results demonstrate a positive correlation between pH and the release of Pyronin Y, indicating that higher pH levels lead to increased release at all time points (Figure 8C). This phenomenon can be attributed to the reduced attractive interactions between the positively charged Pyronin Y molecules and the negatively charged PU−CA hydrogel at an elevated pH (Figure 8D). 

According to our findings from the release study, the results show that regardless of the charge of the loaded compound, the release profiles of the PU−PEI and PU−CA hydrogels demonstrate distinct pH-responsive behaviours. Also, data suggest that when the compound model and the polymeric film have opposite charges (the case for PU−PEI/bromophenol blue and PU−CA/Pyronin Y), the obtained release profiles at different pH values are more significantly altered as compared to their counterparts. The PU−PEI hydrogel exhibits enhanced drug release in acidic pH conditions, especially for positively charged compounds, while the PU−CA hydrogels exhibit increased release in basic pH conditions, especially for negatively charged compounds. These findings provide valuable insights into the pH-dependent characteristics of both hydrogel systems, offering a deeper understanding of their potential applications in drug delivery systems. Specifically, the PU−PEI hydrogel shows promise for acidic wound environments, where the controlled release of therapeutic agents is desired, while the PU−CA hydrogels hold potential for basic wound environments. By modifying the pH-responsive behaviour of these hydrogels, it becomes possible to achieve precise and targeted drug delivery, contributing to breakthroughs in the field of wound healing and therapy.

#### 2.3.2. Step pH Release 

**Figure 9:**
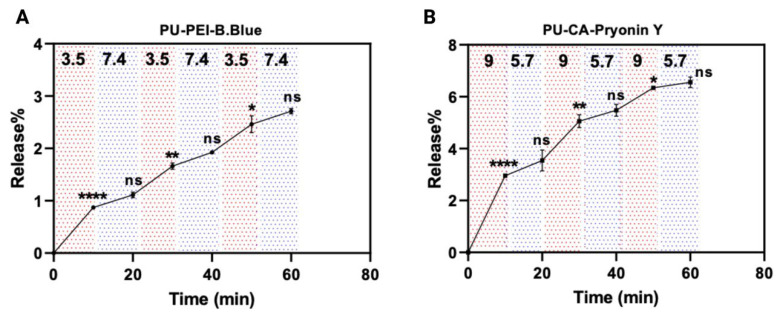
(**A**) The step pH release profile of PU–PEI hydrogel film with bromophenol blue; (**B**) the step pH release profile of PU–CA hydrogel film with Pyronin Y. The *p*-value was used to determine the level of significance for data comparisons, with significance levels represented by asterisks (* *p* < 0.05, ** *p* < 0.01, **** *p* < 0.0001, and ns; not significant).

In order to imitate the dynamic pH changes that can occur in wound environments, the pH-responsive properties of PU−PEI hydrogel with bromophenol blue and PU−CA hydrogel with Pyronin Y were examined using a step pH release test. The surrounding pH was cyclically changed from (3.5) to (7.4) for the PU−PEI hydrogel and from (9) to (5.7) for the PU−CA hydrogel, with each pH condition lasting for 10 min and being repeated for a total of three cycles (Figure 9). The findings showed that both PU−PEI and PU−CA hydrogels displayed sensitivity to variations in the surrounding pH. Specifically, the PU−PEI hydrogel exhibited higher release of bromophenol blue under acidic pH conditions, whereas the PU−CA hydrogel released more Pyronin Y under basic pH conditions. Moreover, it was shown that after every cycle of exposure to pH changes, the responsivity of the polymeric films is reduced until the third cycle, where this sensitivity is completely diminished. Such pattern could be associated with the amount of media that are entrapped within the hydrogel network (due to swelling), which can disrupt the protonation/deprotonation of the corresponding functional groups. Overall, these results emphasise the pH-responsive nature of the hydrogels and their potential suitability for applications in wound healing, where fluctuations in pH can occur.

#### 2.3.3. The Release of Antibiotics 

**Figure 10:**
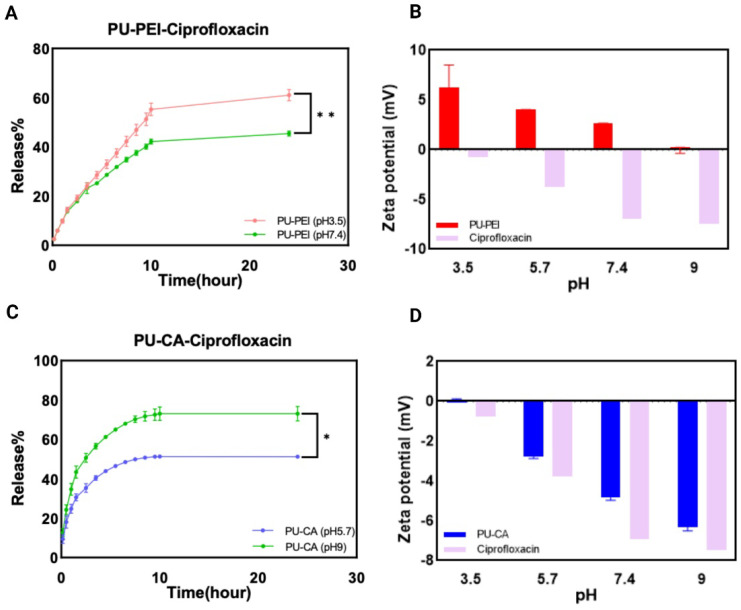
(**A**) The release profile of PU−PEI hydrogel film with ciprofloxacin; (**B**) surface zeta potential measurements of PU−PEI hydrogel films and ciprofloxacin; (**C**) the profile of PU−CA hydrogel film with ciprofloxacin; (**D**) surface zeta potential measurements of PU-CA hydrogel films and ciprofloxacin. The *p*-value was used to determine the level of significance for data comparisons, with significance levels represented by asterisks (* *p* < 0.05, ** *p* < 0.01).

For this section, we conducted in vitro release experiments focusing on an antibiotic that is used to battle infections. The pH-dependent release profile of ciprofloxacin from PU−PEI and PU−CA hydrogels, shown in Figure 10, followed the same trend as compound models. The release profiles of ciprofloxacin from the PU−PEI hydrogel films were examined at two different pH values, (3.5) and (7.4) (Figure 10A). The release of ciprofloxacin from the hydrogel was found to be higher at an acidic pH compared to a neutral pH. This can be attributed to the reduced attraction between the negatively charged drug and the positively charged polymer at an acidic pH. At pH (3.5), approximately 60% of the cumulative ciprofloxacin was released after 24 h, while at pH (7.4), around 40% of ciprofloxacin was released during the same period. The higher release of ciprofloxacin at a lower pH can be explained by the highly positive charge of PU−PEI and minimal charge of ciprofloxacin at an acidic pH, which result in weaker ionic interactions between the amine groups of PEI and the carboxyl groups of ciprofloxacin, facilitating faster drug release compared to higher pH conditions. 

In the case of the PU−CA hydrogel, the release of ciprofloxacin was approximately 22% at pH (5.7) and nearly 35% at pH (9) after 1 h. As time progressed, the disparity between the releases at different pH values notably increased (Figure 10C). After 6 h, the drug release at pH (9) was approximately 1.5 times higher than that at a lower pH. Over a 24 h period, around 48% of ciprofloxacin was released at pH (5.7), while approximately 70% was released at pH (9). At a basic pH, most of the carboxylic groups (-COOH) were ionized, resulting in negatively charged -COO- anions. The repulsion between these anions and negatively charged ciprofloxacin facilitated faster drug release compared to lower pH conditions. Similar to our previous in vitro observations with compound models (Section 2.3.1), PU−PEI films loaded with oppositely charged ciprofloxacin showed a more significant difference in their release profiles over the corresponding pHs when compared to that of PU−CA films. This could imply that attraction forces in these systems can have a more prominent effect on the subsequent release as compared to repulsion forces. In conclusion, the pH-dependent release of ciprofloxacin from both PU−PEI and PU−CA hydrogel primarily relied on the interactions among the polymers and the drug governed by the pH environment. 

### 2.4. In Vitro Antibacterial Activity of the Hydrogel 

**Figure 11:**
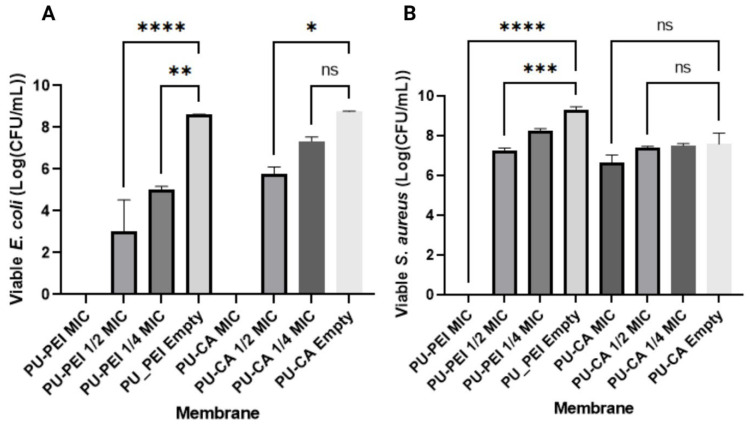
Assessing the viability of (**A**) *E. coli* and (**B**) *S. aureus* that was challenged with membranes loaded with ciprofloxacin compared to empty membranes. One-way ANOVA with Tukey’s multiple comparisons test was applied. Data represent mean ± Std error of mean (SEM), * *p* < 0.05, ** *p* < 0.01, *** *p* < 0.001, **** *p* < 0.0001, ns; not significant; n = 3 biological replicates, with 2 technical replicates each.

Bacterial infection frequently complicates the wound healing process, particularly in chronic wounds, leading to delayed wound repair. Therefore, the development of wound dressings with antibacterial properties has a great clinical significance. These dressings can effectively combat bacterial infections, promoting successful wound healing [36]. *S. aureus* and *E. coli* are prevalent biofilms found in chronic wound tissue, representing Gram-positive and Gram-negative bacterial strains, respectively [37]. In order to assess the antibacterial efficacy of hydrogels, the colony counting method was employed to investigate their antibacterial properties. The minimum inhibitory concentration (MIC) and minimum bactericidal concentration (MBC) of ciprofloxacin were determined beforehand. It was demonstrated that ciprofloxacin can be effective against both *E. coli* and *S. aureus*, exhibiting low MIC values of 0.0313 µg/mL and 1 µg/mL, respectively (Table 1). The MBC values were also low, indicating the antibiotic’s ability to effectively eradicate bacteria at low concentrations. Furthermore, the MBC/MIC ratios ≤ 4 for both bacterial strains demonstrated strong bactericidal activity. The antibacterial properties of PU−PEI and PU−CA hydrogel films containing different concentrations of ciprofloxacin were investigated against *E. coli* and *S. aureus*. The cultured bacteria were tested with hydrogels containing various concentrations of ciprofloxacin (MIC, 1/2 MIC, 1/4 MIC) for 24 h at 37 °C (Figure 11). Subsequently, the medium containing the treated bacteria was diluted and spread onto LB agar plates, followed by incubation for 24 h. The colony-forming units (CFU) on the Petri dishes were quantified to evaluate the antibacterial activity of the hydrogel films. As anticipated, both PU−PEI and PU−CA hydrogels exhibited a favourable antibacterial efficacy against *E. coli* at the MIC concentration, resulting in a complete reduction (100% decrease) in *E. coli* colonies. However, at every other concentration, PU−PEI films seemed to have a more significant antibacterial effect when compared to the empty films. On the other hand, for *S. aureus*, only the PU−PEI hydrogel exhibited a complete elimination at the MIC concentration. When PU−PEI and PU−CA films were compared at equal drug dosages, it was discovered that PU-PEI hydrogel films had a significantly higher antibacterial effect than PU−CA hydrogel films. These data imply that the PU−PEI films exhibited a slower release of the active chemical, particularly at a neutral pH of the culture media (pH 7.4), resulting in an improved antibacterial activity. 

## 3. Conclusions

For the first time, two distinct chemistries were presented for functionalizing HPU with either amine or carboxylic acid groups. We used (i) the aminolysis method to link polyethylenimine (PEI) to the polymer backbone and (ii) the Steglich esterification process to decorate the polymer backbone with succinate acid. Following this, the extensive physiochemical characterization of the synthesized polymers was performed to validate the attachment of selected moieties. Zeta potential studies of modified PU films revealed that amine-modified films (PU−PEI) had a substantially positive charge at an acidic pH (3.5), which gradually diminished with an increasing pH value. Carboxylic-acid-modified films (PU−CA), on the other hand, had no charge at an acidic pH and gradually accumulated more negative charge at a strongly alkaline pH (9). In vitro testing was performed on both types of HPU films for the release of positively and negatively charged model compounds (Pyronin Y and bromophenol blue, respectively). Regardless of compound model charge, the results showed that PU−PEI films released more cargo at an acidic pH, while PU−CA films released more at an alkaline pH. Furthermore, a step pH release test demonstrated that these films were responsive even after three cycles of pH adjustments. To assess their feasibility for chronic wound drug administration, these films were loaded with ciprofloxacin (an antibiotic for both Gram-negative and Gram-positive aerobic bacteria) and then tested in vitro on *Staphylococcus aureus* (*S. aureus*) and *Escherichia coli* (*E. coli*). At the same medication dosages, PU−PEI films had considerably greater antibacterial activity than PU−CA films. These findings revealed that, at the neutral pH of the culture media (7.4), PU−PEI films had a delayed release profile, resulting in an enhanced antibacterial action. Overall, the chemically modified HPUs showed promise as wound dressings capable of releasing antibiotics in response to the pH of infected wounds. 

## 4. Materials and Methods 

### 4.1. Materials 

*Staphylococcus aureus* (ATCC 25213) and *Escherichia coli* (ATCC 25922) were purchased from the American Type Culture Collection (ATCC, Manassas, VA, USA). The following materials were sourced from Sigma Aldrich (St. Louis, MO, USA): Dulbecco’s Phosphate Buffered Saline, Sodium Chloride, Potassium chloride (KCl), Hydrochloric Acid (HCl), Sodium hydroxide (NaOH), Dimethylformamide (DMF), polyethyleneimine (PEI), triethylamine, 4-dimethylaminopyridine (DMAP), succinic anhydride, bromophenol blue, Pyronin Y, ciprofloxacin, and LB agar plates. HydroMed D3 (AdvanSource, Wilmington, MA, USA; referred to as PU hereafter) was used as the base polyurethane material. 

### 4.2. Synthesis of PU−PEI hydrogel

PU−PEI was synthesized using the aminolysis mechanism [32], by reacting PU with low-molecular-weight branched polyethyleneimine (M_w_ = 800). Briefly, 10 g of PU was dissolved in 100 mL of a solvent (DMF) and subsequently 5 mL of a base catalyst (triethylamine) was added to the solution. Next, 5 mL of PEI was diluted in methanol and added to the reaction mixture. The temperature was set at 45 °C and the reaction was allowed to progress over 48 h. Lastly, the polymer solution was precipitated in excess diethyl ether, and washed in multiple successive rounds with methanol, ethanol, and water. The clean polymer was at last lyophilized overnight and kept in closed containers for further processing. 

### 4.3. Synthesis of PU−CA Hydrogel

PU−CA was synthesized using a modified Stiglich esterification mechanism [33], by reacting PU with succinic anhydride. Briefly, 10 g of PU was dissolved in 100 mL of a solvent (DMF) and subsequently 500 mg of the catalyst 4-dimethylaminopyridine (DMAP) was added to the reaction mixture. Next, 5 g of succinic anhydride was added to the mixture, the temperature was set at 65 °C, and the reaction was allowed to progress for 24 h. Lastly, the polymer solution was precipitated in excess diethyl ether, and washed in multiple successive rounds with methanol, ethanol, and water. The clean polymer was at last lyophilized overnight and kept in closed containers for further processing. 

### 4.4. Preparation of Hydrogel Films

The film-casting method was used to prepare hydrogel films. Accordingly, a 5% polymer solution was prepared in the solvent system containing 90% ethanol and 10% water (*v*/*v*). The solution was casted in Petri dishes and dried overnight at room temperature. Upon rehydration, intermolecular hydrogen bonds generated by urethane and urea groups along the polymer chains would create physical crosslinking, hence preventing the dissolution of modified PU and forming highly stable and elastic hydrogels. For preparing model-compound-loaded films, a concentration of 0.5 mM of either bromophenol blue or Pyronin Y was added to the polymer solution before casting. Ciprofloxacin-loaded films were prepared using 1 mg/mL of the drug in the polymeric solution. Dried hydrogel films were kept away from humidity in enclosed containers. 

### 4.5. Physicochemical Characterization of Polymers

Fourier transform infrared (FT-IR) spectra were obtained with a Thermo (Boston, MA, USA) Nicolet 6700-attenuated total reflectance FT-IR spectrometer with a frequency range of 550–4000 cm^−1^. The analysis was performed with a resolution of 4 cm^−1^, and the output signal was recorded as absorbance. Analytical-size-exclusion chromatography (SEC) was performed using a Shimadzu Prominence UFLC (ultra-fast liquid chromatography) system fitted with a Shim-pack SEC-800DP guard column followed by two in-series Phenogel columns (5 mm, 104 Å and 105 Å). The system eluent was HPLC-grade dimethylacetamide (DMAc) with BHT/LiBr at 0.05 wt%, eluting at a flow rate of 1 mL min^−1^. The column assembly was incubated at 50 °C, and retention times were calibrated using PMMA standards from PSS. Polymers were dissolved and passed through a 400 nm nylon filter prior to injection. A thermogravimetric analysis (TGA) was conducted using a TA Q5000 instrument. Approximately 8–10 mg of each sample was weighed and placed in a platinum pan, which was then heated from 30 °C to 600 °C at a rate of 15 °C per minute under a nitrogen (N2) atmosphere with a flow rate of 20 mL/min. Differential scanning calorimetry (DSC) was investigated with a TA Q1000 instrument in the temperature range of −40 to 200 °C at a rate of 10 °C per minute under a nitrogen atmosphere. Proton nuclear magnetic resonance (1HNMR-Bruker 800 MHz) was employed to investigate the successful synthesis of PU-PEI and PU-CA by dissolving them in a mixture solvent containing 90% ethanol and 10% water (*v*/*v*). The elemental composition at the surface of each polyurethane sample was analysed using X-ray Photoelectron Spectroscopy (K-alpha + XPS, Thermo Fisher Scientific). Samples were fixed to a standard stage and loaded into the instrument. The XPS experiment was performed using the connected software (Avantage Data System, Version 2022, Thermo Fisher Scientific); the X-ray spot gun was set to 400 μm, the flood gun was turned on, and the spectra obtained for each sample included C1s, O1s, and N1s orbitals, and a survey of all binding energies. The resulting data were analysed using the Avantage software, Version 2022, wherein the peaks identified in the spectra were deconvoluted and correlated to specific bonds for each element. Oxygen and nitrogen counts were normalised against the C-C bond peak to aid comparison in elemental composition between samples. The rheological properties of the hydrogels were assessed at room temperature using an Anton Paar MCR 301 system in parallel plate geometry (250 mm disk, 0.5 mm measuring distance). An oscillation test was performed on polymer solutions (5% *w*/*v*) at a strain rate of 0.1% in the frequency range 0.1–10 HZ. Shear tests were performed with a shear rate ranging from 1 to 100 s^−1^ to determine the viscosity of polymers. 

### 4.6. Physicochemical Characterization of Hydrogel Films

The mechanical properties of the films were evaluated using an INSTRON 5943 (USA) mechanical tester. Rectangular samples with dimensions of 10 mm in width, 20 mm in gauge length, and a thickness of 0.1 mm were used for the test. The tests were conducted at a rate of 60 mm/min with at least three replicates. The zeta potential of the membrane surfaces was evaluated using an electrokinetic analyser (SurPASS 3, Anton-Paar). Two membrane coupons measuring 20 mm × 10 mm were affixed to the sample holders of an adjustable gap cell with a 100 μm gap size. The measuring solution consisted of an aqueous 0.01 M KCl solution, with 0.05 M of HCl and 0.05 M of NaOH used for pH adjustment. Zeta potential measurements were taken sequentially at pH (3.5), (5.7), (7.4), and (9), and were computed using the Helmholtz–Smoluchowski equation in the SurPASS 3 software, Version 2020. To obtain the average zeta potential value with standard deviation, a minimum of five measurements were taken. The surface charge of the particles was evaluated via zeta potential measurements using a Malvern zetasizer Nano ZS (Malvern Instruments, Malvern, UK), with a field strength of 20 V/cm applied. The zetasizer Nano assessed the electrophoretic mobility of the particles, and the resulting data were converted to the zeta potential using the Helmholtz–Smoluchowski equation incorporated into the Malvern zetasizer software, Version 2022. Since the zeta potential of particles is influenced by the dispersion medium, measurements were performed in both double-distilled water adjusted to 50 μS/cm using a 0.9% NaCl solution and in the original dispersion medium. To determine the swelling ratio of the samples, they were immersed in separate phosphate-buffered saline (PBS) solutions of pH (3), (5.7), (7.4), and (9) for 72 h at 32 °C. The pH of solutions was adjusted using 0.1 of HCl and 0.1 M of NaOH, as needed. The mass of each sample was measured before and after submersion, and the swelling ratio was calculated using the following equation: swelling ratio = $\left(\frac{W_{s}-W_{d}}{W_{d}}\right)\times 100$, where *W_d_* is the weight of the dry sample before immersion, and *W_s_* is the weight of the submerged sample after 72 h. The test was repeated three times, and average values were used for the representation of data.

### 4.7. In Vitro Release Studies

The release of bromothymol blue (BTB), Pyronin Y, and ciprofloxacin from the hydrogels was assessed in PBS. Specifically, 15 mg of the samples were separately immersed in 500 μL of PBS solutions of pH (3.5), (5.7), (7.4), and (9), and maintained at 32 °C. The absorbance was monitored for BTB, Pyronin Y, and ciprofloxacin at 590 nm, 540 nm, and 320 nm, respectively, using a UV-spectrometer (SpectraMAX M3, San Jose, CA, USA) at predetermined intervals. To ensure a continuous release, 100 μL of the solution was withdrawn each time and replaced with an equal volume of a fresh PBS solution. All experiments were performed in triplicate. The amount of BTB, Pyronin Y, and ciprofloxacin released was quantified using a calibration curve generated with various concentrations of BTB, Pyronin Y, and ciprofloxacin prepared in PBS. The step pH release test was conducted for PU−PEI hydrogel with bromophenol blue and PU−CA hydrogel with Pyronin Y. In total, 15 mg of the samples were immersed in 500 μL of PBS solutions with pH cyclically altered from (3.5) to (7.4) for the PU−PEI hydrogel and from (9) to (5.7) for the PU−CA hydrogel. Each pH condition lasted for 10 min, repeated for a total of 3 cycles with triplicate.

### 4.8. In Vitro Antibacterial Activity Assays

The antibacterial activity of the hydrogels was evaluated using the colony counting method with *S. aureus* (Gram-positive) and *E. coli* (Gram-negative) as bacterium models. Overnight cultures of both bacteria were prepared in a sterilized nutrient broth at 37 °C under shaking at 160 rpm. The hydrogel samples were exposed to UV light for 1 h and placed under a hood overnight. Then, bacterial suspensions of 1 × 10^6^ CFU mL^−1^ were added to 96-well plates and incubated at 37 °C for 24 h. Subsequently, the solutions in each well were diluted 100,000 times and plated onto LB agar plates, which were then incubated at 37 °C for 24 h for *E. coli* and *S. aureus*. The number of colonies forming units (CFUs) on the LB agar plates was then quantified. Three biological replicates were tested with 2 technical replicates.

### 4.9. Statistical Analysis

The mean ± standard error was used to report the data. The statistical analyses were performed using GraphPad Prism 9.5.1 software, Version 2023, utilizing a one-way analysis of variance (ANOVA) and two-way ANOVA. Each test included nine samples. The *p*-value was used to determine the level of significance for data comparisons, with significance levels represented by asterisks (* *p* < 0.05, ** *p* < 0.01, *** *p* < 0.001, **** *p* < 0.0001, ns; not significant).

## Figures and Tables

**Table 1:** Assessing the antimicrobial susceptibility of *E. coli* and *S. aureus* to free ciprofloxacin and EtOH/water blend. Data represents mean; n = 3 biological replicates, with 2 technical replicates each.

	Ciprofloxacin			EtOH Water (Control)		
**Bacteria**	**MIC ** **(µg/mL)**	**MBC ** **(µg/mL)**	$\frac{MBC}{MIC}$	**MIC ** **(%)**	**MBC ** **(%)**	$\frac{MBC}{MIC}$
*E. coli*	0.0313	0.0313	≤4	0.063	12.5	≤4
*S. aureus*	1	1	≤4	12.5	12.5	≤4

## Data Availability

Data is available upon request.

## Supplementary Materials

The following supporting information can be downloaded at: https://www.mdpi.com/article/10.3390/gels9080611/s1, Figure S1: HNMR spectra of (A) PU; (B) PU–PEI; and (C) PU–CA in an ethanol d4–D2O mixed solvent system.; Figure S2: XPS spectra of polymers including the survey scan (top) and narrow scan of C1s, N1s, and O1s (from right to left, respectively); Figure S3: (A) Viscosity measurement of 5% (*w*/*v*) of PU, PU–PEI, and PU–CA; (B) storage modulus (E’) and loss modulus (E”) of PU, PU–PEI, and PU–CA; Figure S4: (A) The swelling test of PU–PEI hydrogel films at pH (3.5), (5.7), and (7.4); B) the swelling test of PU–CA hydrogel films at pH (5.7), (7.4), and (9); Table S1: Molecular weight of PU, PU–PEI, and PU–CA.
