# Supplementary material for: Synthesis and Evaluation of Functionalized Polyurethanes for pH-Responsive Delivery of Compounds in Chronic Wounds
Source: Gels. 2023 Jul 28;9(8):611. doi: 10.3390/gels9080611 (PMC10454082; doi:10.3390/gels9080611)
Supplement: Supplementary file 1 [file gels-09-00611-s001.zip › gels-2520375-supplementary.pdf]

gels

Article

Synthesis and evaluation of functionalized polyurethanes for pH-responsive delivery of compounds: Potential dressing materials for chronic wounds

[1] Faculty of Engineering, School of Chemical and Biomolecular Engineering, The University of Sydney, Sydney, New South Wales, Australia.
[2] Key Centre for Polymers and Colloids, School of Chemistry, The University of Sydney, Sydney, NSW 2006, Australia.
[3] Sydney Institute for Infectious Diseases, The University of Sydney, Australia.
[4] Nano Institute (Sydney Nano), The University of Sydney, Sydney, New South Wales, Australia.
* Correspondence: Sepehr.talebian@sydney.edu.au (S.T) and Sina.naficy@sydney.edu.au (S.N)

Citation: Li, Z.; Crago, M.; Schofield, T.; Zeng, H.; Vyas, H.K.N.; Müllner, M.; Mai-Prochnow, A.; Farajikhah, S.; Naficy, S.; Dehghani, F.; et al. Synthesis and evaluation of functionalized polyurethanes for pH-responsive delivery of compounds: Potential dressing materials for chronic wounds. *Gels* **2023**, *volume number*, x. https://doi.org/10.3390/xxxxx

Academic Editor(s):

Received: date
Revised: date
Accepted: date
Published: date

Copyright: © 2023 by the authors. Submitted for possible open access publication under the terms and conditions of the Creative Commons Attribution (CC BY) license (https://creativecommons.org/licenses/by/4.0/).

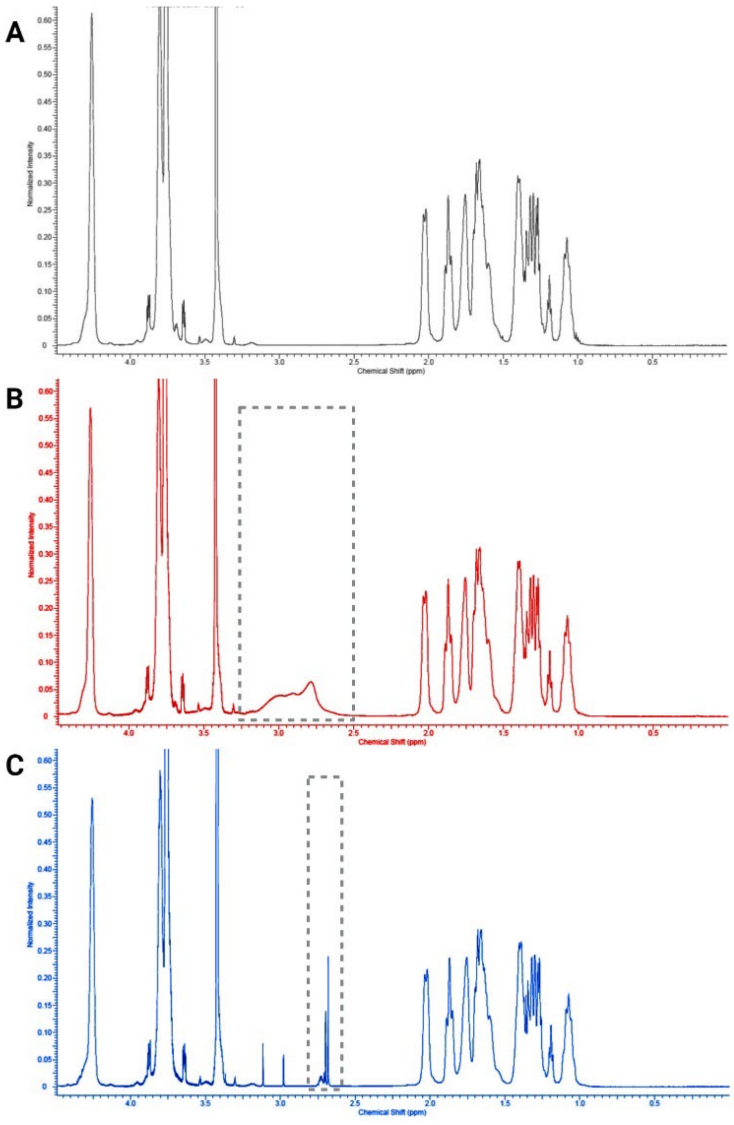

Figure S1. HNMR spectra of A) PU; B) PU−PEI; C) PU−CA in ethanol d4−D2O mixed solvent system.

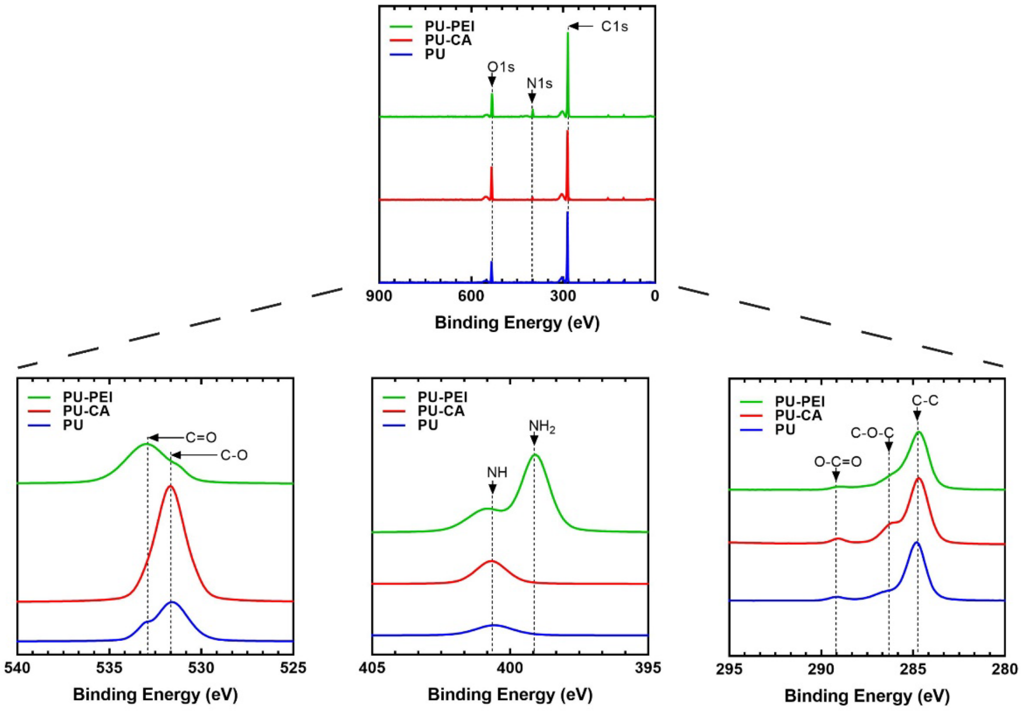

Figure S2. XPS spectra of polymers including survey scan (Top) and narrow scan of C1s, N1s, and O1s (from right to left, respectively).

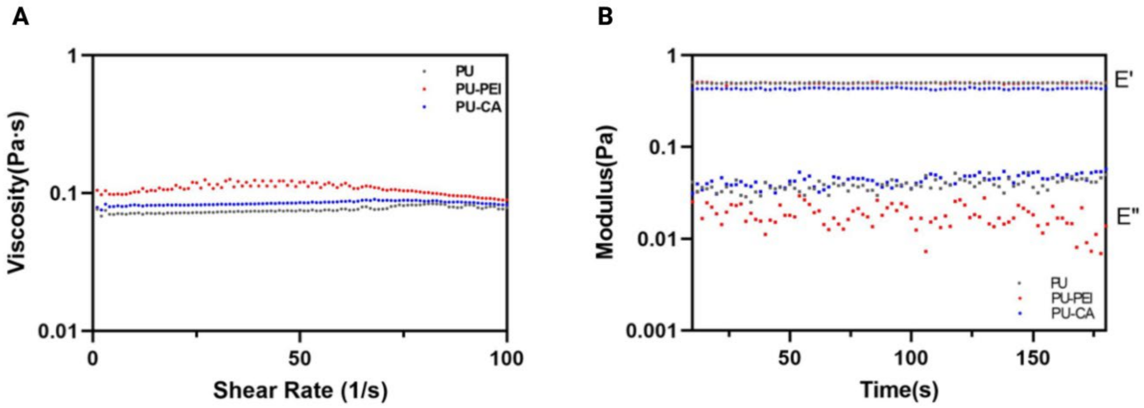

Figure S3. A) Viscosity measurement of 5% (w/v) of PU, PU−PEI and PU−CA; B) Storage modulus (E′) and loss modulus (E″) of PU, PU−PEI and PU−CA.

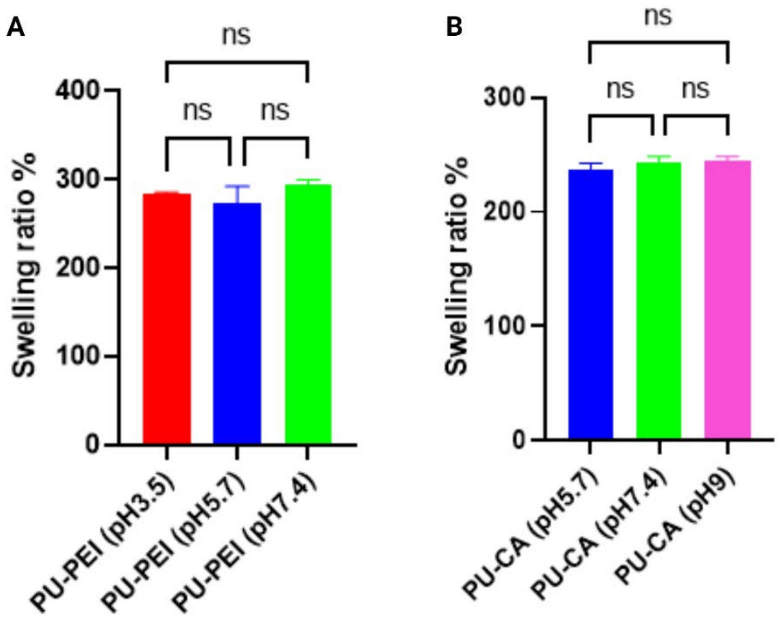

Figure S4. A) The swelling test of PU−PEI hydrogel films at pH (3.5), (5.7) and (7.4); B) The swelling test of PU−CA hydrogel films at pH (5.7), (7.4) and (9).

Table S1. Molecular weight of PU, PU-PEI and PU-CA.

	**PU**	**PU−PEI**	**PU−CA**
M_n	172800 Da	106800 Da	101300 Da
M_w	332300 Da	267200 Da	223100 Da
M_w/M_n	1.92	2.50	2.20
